# Supplementary material for: Talent Selection Based on Sport-Specific Tasks Is Affected by the Relative Age Effects among Adolescent Handball Players
Source: Int J Environ Res Public Health. 2021 Oct 29;18(21):11418. doi: 10.3390/ijerph182111418 (PMC8583324; doi:10.3390/ijerph182111418)
Supplement: Supplementary file 1 [file ijerph-18-11418-s001.zip › ijerph-1399379-supplementary.pdf]

Supplementary material to manuscript ID: ijerph-1399379

Talent selection based on sport-specific tasks is affected by the relative age effects among adolescent handball players

DESCRIPTION OF SELECTION TASKS

Handball-specific tasks

Task 1 (dribbling-shooting) (illustration 1)

Starting position: Next to a cone placed at the 9m line holding a ball

Task: The players had to run along with the court by dribbling the ball and execute a jump-shot on goal against a goalkeeper, then run back to 7m, pick up another ball, dribble the ball around cones placed in zig-zag arrangement using always the outer arm when dribbling, and perform a jump-shot from the free-throw line, then finish with running back to initial position.

Time recording: From start until touching the free-throw line after the last run.

Repetitions: two times (with short rest in-between)

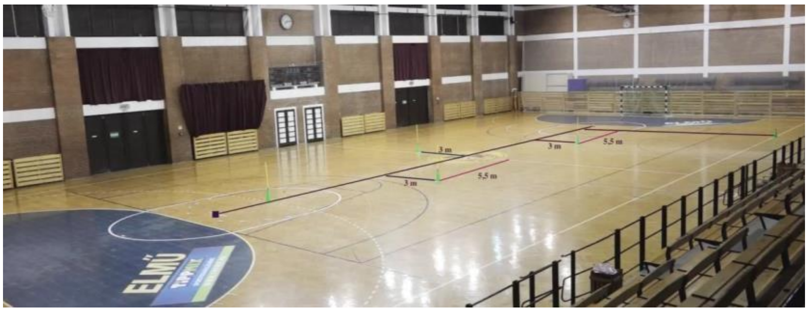

Illustration 1.

Task 2 (defensive footwork) (illustration 2)

Starting position: By the external side of a cone placed at the right inside defensive position.

Task: On starting signal horizontal and lateral defensive steps around nine cones placed diagonally three meters from each other.

Time recording: From starting signal until touching the last cone.

Repetitions: Two times (with short rest in-between)

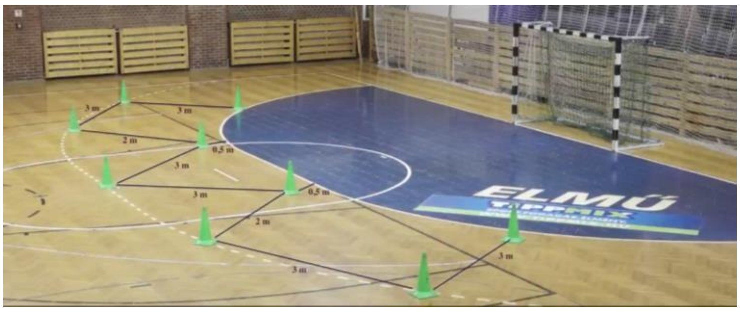

Illustration 2.

Position-specific technical tasks

BACKCOURTS

Task 1 (girls) (illustration 3)

Starting position: By the outer side of a cone placed at 12m from the goal holding a ball (red cone on illustration 3).

Task: Run to the 9m line, pass the ball to a fellow player standing at the opposite side backcourt position, then run back to 12m, pick up another ball, dribble once and execute a jump-shot on goal corner shooting target and then run to the center line.

Time recording: At county level no time recording. At regional level time recording from the start until the last run to the center line after the fifth attempt.

Repetitions: At county level five repetitions (with short rest in-between). At regional level five repetitions continually with no rest in-between repetitions.

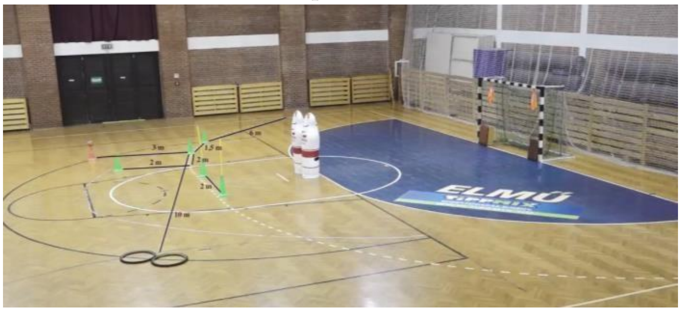

Illustration 3.

Task 1 (boys) (illustration 4)

Starting position: By the outer side of a cone placed at 12m from the goal holding a ball.

Task: Run to the free-throw line, execute a body feint and pass by the throwing arm side and throw the ball with a jump to an air body placed at the opposite corner. Then passing through two cones at 9m run back to 12m, pick up a ball from the floor, dribble once and perform a shot on goal corner shooting target: three times with jump, one time with leaning back shot, and one time with underarm shot. Then run back to the center line.

Time recording: At county level no time recording. At regional level time recording from the start until the last run to the center line after the fifth attempt.

Repetitions: At county level five repetitions (with short rest in-between). At regional level five repetitions continually with no rest in-between repetitions.

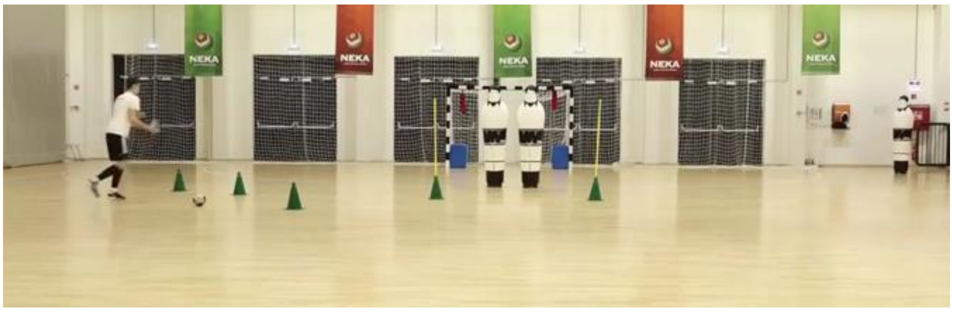

Illustration 4.

Task 2 (illustration 5).

Starting position: 12m from the goal at side backcourt position holding a ball.

Task: Run to the free-throw line, pass the ball to a fellow player standing at the opposite side backcourt position, then run back and pass around a cone at 11m, receive the ball, run to 9m, perform a shot feint in front of an air body,

dribble once to 7m, perform a body feint next to another air body, execute a fly shot on goal against a goalkeeper, and finish with running back to the center line.

Time recording: At county level no time recording. At regional level time recording from the start until the last run to the center line after the fifth attempt.

Repetitions: At county level five repetitions (with short rest in-between). At regional level five repetitions continually with no rest in-between repetitions.

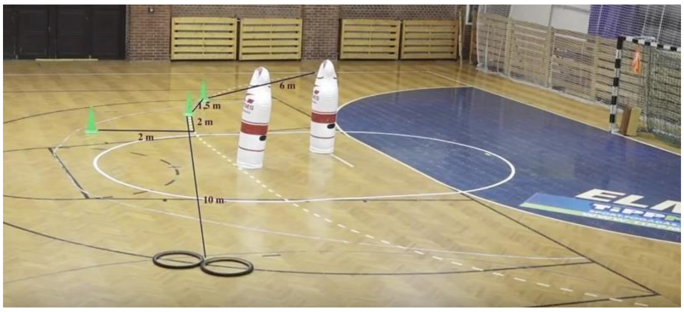

Illustration 5.

WINGS

Task 1 (illustration 6).

Starting position: Far right/left position without a ball.

Task: Start without a ball, receive the ball from a fellow player while running into the area between two air bodies placed two meters from each other along the 6m line, return the ball and run back to the initial position, receive the ball again, execute a jump-shot on goal by the external side of the outer air body, and run to the junction of the side and center lines

Time recording: At county level no time recording. At regional level time recording from the start until the last run to the center line after the fifth attempt.

Repetitions: At county level five repetitions (with short rest in-between). At regional level five repetitions continually with no rest in-between repetitions.

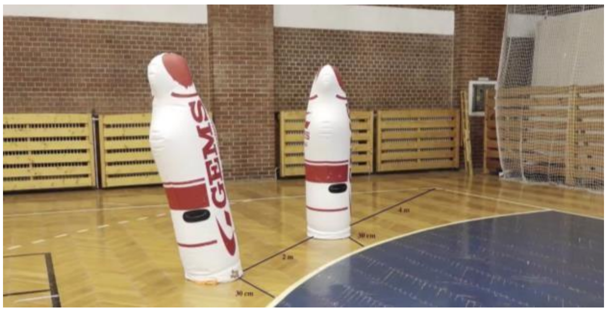

Illustration 6.

Task 2 (illustration 7).

Starting position: At the junction of the side and free-throw lines holding a ball.

Task: Run with the ball into the area between two air bodies placed two meters from each other along the 6m line, pass the ball to a fellow player standing at the same side backcourt position, run back to starting position, attack the air bodies, receive the ball and by avoiding the external air body execute a jump-shot on goal, then run back to the center line.

Time recording: At county level no time recording. At regional level time recording from the start until the last run to the center line after the fifth attempt.

Repetitions: At county level five repetitions (with short rest in-between). At regional level five repetitions continually with no rest in-between repetitions.

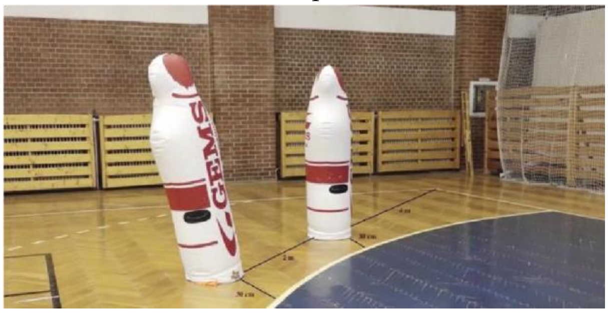

Illustration 7.

PIVOTS

Task 1 (illustration 8).

Starting position: At the pivot position in front of an air body without a ball.

Task: Receive the ball above the head from the center backcourt, rotate to the opposite side of the throwing arm and execute a shot with falling. Next, stand in front of an air body placed at the side backcourt position, receive the ball at chest level from the left backcourt, rotate by the same side of the throwing arm and execute a shot with falling. Next, stand in front of an air body placed at the other side backcourt position receive the ball from the right backcourt, rotate to the opposite side of the throwing arm and execute a shot with falling.

Time recording: No time recording at any selection level.

Repetitions: At county level 2x3 shots (with short rest after the third shot). At regional level 2x6 shots (with short rest after the sixth shot).

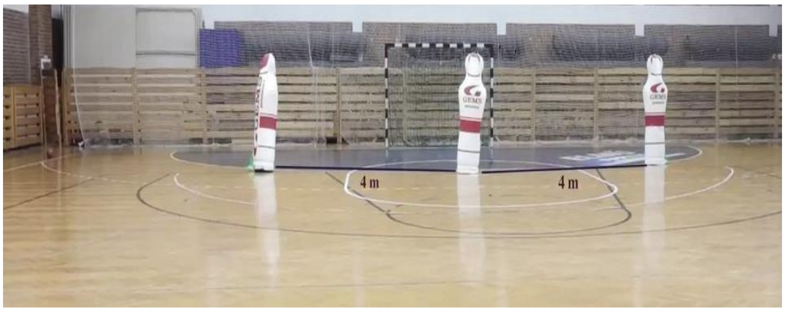

Illustration 8.

GOALKEEPERS

Task 1

Starting position: On the middle of the goal line

Task: On signal, touch the goalpost at the following order: left low, right up, left up and right low, then slide to the left low corner, run to the 4m line, pick up and pass the ball to a fellow player standing at the center line.

Time recording: From starting signal until the fellow player catches ball.

Repetitions: Two repetitions (at the 2nd repetition the goalkeeper begins the task at the right side).

Evaluation and scoring

	Tasks (points)	Criteria (points)	Description
**On-game performance**			
	5		Offensive skills<br>Offensive footwork and attack actions to the goal with and without the ball according to playing positions. Use of actions with players (movement in space with and without the ball, screenings). Counterattacks efficacy (passing, shooting accuracy, position changes). Individual offensive actions (shooting strength, mobility, agility, decision making)
	5		Defensive skills<br>Position changes during the game, good use of lateral and horizontal movements. Use of one-to-one defense. Contacts and good blocking technique. Two-to-two defensive actions, movement in space, pressing and zone defense. Positioning during return to defense. Individual defensive actions (body type, action specialist, appropriate aggressivity/determination).
total points	10		
**Handball-specific generic skil**ls			
dribbling	5		Technical execution 0-1 points, time duration 0-4 points
defensive footwork	5		time duration 0-5 points
**Position-specific technical drills**			
Backcourts			
Task 1	10	3	accuracy of long pass
			one point for hitting the air body above the hip
		2	hit the shooting target
		5	technical execution (5x)
			run-up
			footwork
			shooting (3x with jump + 1x with leaning back,1x under arm)
Task 2	10	2	accuracy of long pass
		3	goal scoring (against a goalkeeper)
		5	technical execution (5x)
			walking mistake
			two phase movement dynamics
			shot feint (2x to one direction, 3x to the other direction)
total points	20		

Wings			
Task 1	10	3	goal scoring (against a goalkeeper)
		5	technical execution (5x)
			technical mistake, step within the goal area
			accuracy of passing and catching the ball
			direction of diving
		1	diving, rolling, sliding out
		1	shot feint while flying, landing
Task 2	10	3	goal scoring (against a goalkeeper)
		5	technical execution of feints (5x)
			walking mistake
			two phase movement dynamics
			feint 2x to one direction, 3x to the other direction
		1	diving, rolling, sliding out
		1	shot feint while flying, landing
total points	20		
Pivots	20	6	goal scoring (against a goalkeeper)
1.		6x2	technical execution (2x3)
			technical mistake, step within the goal area
			landing
			jump direction
			leaning or diving
		2	catching the ball with one hand
total points	20		
Goalkeepers	20		
1.		5	defend shots from backcourts
		5	defend shots from pivots
		5	defend shots from wings
total points	20		
TOTAL	40		
